# A Simple *in situ* Assay to Assess Plant-Associative Bacterial Nitrogenase Activity

**DOI:** 10.3389/fmicb.2021.690439

**Published:** 2021-06-23

**Authors:** Timothy L. Haskett, Hayley E. Knights, Beatriz Jorrin, Marta D. Mendes, Philip S. Poole

**Affiliations:** Department of Plant Sciences, University of Oxford, Oxford, United Kingdom

**Keywords:** nitrogen fixation, symbiosis, diazotroph, acetylene reduction assay, endophyte

## Abstract

Assessment of plant-associative bacterial nitrogen (N) fixation is crucial for selection and development of elite diazotrophic inoculants that could be used to supply cereal crops with nitrogen in a sustainable manner. Although diazotrophic bacteria possess diverse oxygen tolerance mechanisms, most require a sub 21% oxygen environment to achieve optimal stability and function of the N-fixing catalyst nitrogenase. Consequently, assessment of N fixation is routinely carried out on “free-living” bacteria grown in the absence of a host plant and such experiments may not accurately divulge activity in the rhizosphere where the availability and forms of nutrients such as carbon and N, which are key regulators of N fixation, may vary widely. Here, we present a modified *in situ* acetylene reduction assay (ARA), utilizing the model cereal barley as a host to comparatively assess nitrogenase activity in diazotrophic bacteria. The assay is rapid, highly reproducible, applicable to a broad range of diazotrophs, and can be performed with simple equipment commonly found in most laboratories that investigate plant-microbe interactions. Thus, the assay could serve as a first point of order for high-throughput identification of elite plant-associative diazotrophs.

## Introduction

Exploiting diazotrophic bacteria that reduce atmospheric dinitrogen (N_2_) into ammonia (NH_3_^+^) as inoculants of cereal crops has great potential to alleviate current inputs of environmentally deleterious fertilizer nitrogen (N) in agricultural systems to establish more sustainable crop production (Santos et al., 2019). Many diazotrophic strains have been isolated that colonize the root compartments (rhizosphere, rhizoplane, and endosphere) of cereals (Rosenblueth et al., 2018), but it remains unclear as to which strains are best suited for agriculture. “Elite” inoculants should ideally (a) competitively colonize and persist in plant root compartments to exert their beneficial effects, (b) exhibit some degree of interactive specificity with the target host to prevent promiscuous growth promotion of non-target species, and (c) fix and release large quantities of N for assimilation by the plant (Haskett et al., 2020). Although no natural bacteria have been categorically demonstrated to satisfy these three criteria, targeted selection and genetic engineering programs are currently underway to assist in the development of elite inoculant strains (Geddes et al., 2015; Rosenblueth et al., 2018; Bueno Batista and Dixon, 2019; Bloch et al., 2020; Li and Chen, 2020; Ryu et al., 2020).

Assessment of plant-associative bacterial N fixation is central to the selection and development of elite inoculant strains and is typically carried out using ^15^N incorporation assays (Chalk, 2016; Doty et al., 2016; Herridge and Giller, 2016; Van Deynze et al., 2018). While these assays can be highly accurate, they are also laborious and must be performed on both symbiotic partners if measurements of total N-fixed are required. Natural diazotrophic bacteria typically release little of their fixed N when cultured in N-fixing conditions (Ferdinandy-van Vlerken et al., 1991; Machado et al., 1991; Colnaghi et al., 1997; Bueno Batista and Dixon, 2019), but transfer of fixed N to plants has be detected (Chalk, 2016), presumably following lysis of bacterial cells. As an alternative to ^15^N incorporation assays, nitrogenase activity can be assessed using acetylene reduction assays (ARA), which rely on the use of gas chromatography (GC) to monitor the reduction of acetylene (C_2_H_2_) to ethylene (C_2_H_4_) (Hardy et al., 1968). Monitoring this alternative reaction provides a rapid strategy to measure total nitrogenase activity independently of the fate of fixed N and can serve as a proxy for measurements of N fixation.

Due to the oxygen-sensitive nature of nitrogenase and subsequently N fixation, diverse oxygen tolerance mechanisms have evolved in diazotrophic bacteria (Lery et al., 2010). Some, such as *Azotobacter vinelandii* can fix N optimally in an external environment of 21% oxygen (air) (Sabra et al., 2000). However, most diazotrophs require a sub 21% oxygen environment that may not be conducive to plant growth (Halbleib and Ludden, 2000). Measurements of nitrogenase activity and N-fixation are therefore routinely performed in the absence of a host plant. Critically, these measurements may not reflect activity in the rhizosphere where the availability and forms of nutrients such as carbon (C) and N, which are key regulators of N fixation (Little et al., 2000; Little and Dixon, 2003; Ninfa and Jiang, 2005; Bueno Batista and Dixon, 2019), may vary widely. As we are continually isolating novel putative plant-associative diazotrophic bacteria from the environment, a high-throughput assay to confirm and compare nitrogenase activity in an environment more reflective of plant root and rhizosphere would be of significant value to assess their use as cereal inoculants.

**FIGURE 1:**
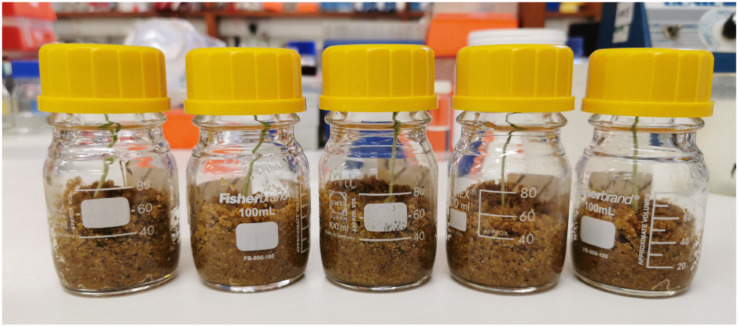
*In situ* ARA vessels. Schott bottles contain 6-dpi barley plants with a headspace atmosphere of 1% O_2_ and 10% C_2_H_2_.

Here, we present a simple *in situ* ARA to assess nitrogenase activity in diazotrophic bacteria occupying the root system of the model cereal barley (Figure 1). We demonstrate that the assay is highly reproducible, rapid, applicable to genetically diverse diazotrophs, and requires minimal equipment commonly found in laboratories investigating plant-microbe interactions.

## Materials and Equipment

• 7% (v/v) NaOCl
• 70% (v/v) ethanol
• 0.9% (v/v) water agar
• Phosphate buffered saline (PBS)
• 100 mL Schott bottles
• Plastic screw caps with aperture
• Rubber septums
• N_2_ gas cylinder
• Acetylene gas cylinder
• Dried industrial yellow fire sand (Cat. No BFS1, sourced from www.thesafetycenter.co.uk)
• 20 mL syringes
• 1 mL syringes
• 23–24-gauge syringe needles
• UMS media (Brown and Dilworth, 1975; Poole et al., 1994) or other bacterial growth media
• Nitrogen and carbon-free rooting solution (CaCl_2_⋅2H_2_0 2.67 mM, KCl 276 μM, MgSO_4_⋅7H_2_O 2.13 mM, Fe EDTA 26.67 μM, H_3_BO_3_ 93.33 μM, MnCl_2_⋅4H_2_O 24 μM, ZnCl_2_ 2.13 μM, Na_2_MoO_4_⋅2H_2_O 1.33 μM, CuSO_4_⋅5H_2_O 0.8 μM, KH_2_PO_4_ 1.33 g/L, Na_2_HPO_4_ 1.52 g/L).
• Spectrophotometer (capable of OD600_*nm*_ measurements)
• Gas chromatograph.

## Methods

### *In situ* ARA Protocol

#### Prepare Inoculant

Prepare inoculants for *in situ* ARAs by streaking single colonies of bacteria onto 10 mL agar slopes in 30 mL universal tubes and incubating for 1–2 days. Once cultures are grown, wash cultures from the slopes three times with PBS to remove residual N and resuspend in N/C-free UMS media (Poole et al., 1994) at OD_600 nm_ 0.001–1.

#### Germinate Seedlings

Surface sterilizes barley seeds (golden promise was used for experiments in this study) by submersion in 70% ethanol for 2 min and 7% NaOCl for 2 min, then rinse thoroughly in sterile water. Germinate seedlings on 0.9% water agar at room temperature in the dark for 2-days or until radicles of ∼1 cm appear.

#### Prepare Assay Bottles

To house the barley seedlings, fill 100 mL Schott bottles (the true volume is 130 mL) with 50 g of industrial grade sand 15 mL of N-free and C-free rooting solution, then autoclave.

#### Sew Seedlings and Inoculate

Sew one pre-germinated seedling into each bottle and immediately inoculate with 2 mL of washed bacterial suspension in UMS. Cover the openings of Schott bottles with sterile cling film and placed in a growth chamber (a 23°C 16 h light / 21°C 8 h dark cycle was used in these experiments). Inclusion of uninoculated control plants in the experiment can be useful to assess potential contamination.

#### Adjust O_2_ in the Headspace

At 6-dpi, place Schott bottles in a controlled atmosphere cabinet adjusted to 1% O_2_ by flushing with N_2_ gas. Leave for 1 h then seal the bottles using screw caps with a rubber septum covering the aperture. Note that other strategies used to adjust O_2_ concentration may be equally viable.

#### Initiate the ARA

Replace 10% of the headspace atmosphere (16.5 mL of air) with C_2_H_2_ (13 mL) using a 20 mL syringe with 22–24 gauge needle. Return plants to the growth chamber.

#### Measure the Evolution of C_2_H_4_

Using a 1 mL syringe, extract aliquots of the headspace atmosphere at 12–24-h intervals and measure the evolution of C_2_H_4_ by gas chromatography. In this study, a PerkinElmer, Clarus 480 gas chromatograph equipped with a HayeSep^®^ N (80–100 MESH) 584 column was used. Oven temperature was 100°C with flame ionization detector (FID) temperature set to 150°C. Flow rate of the nitrogen carrier gas was 20 mL/min.

#### Analyze Data

Total C_2_H_4_ production at each timepoint is calculated as previously described (Herridge and Giller, 2016) by deriving the fraction of the C_2_H_4_ peak area compared to C_2_H_2_, and multiplying this value by the number of C_2_H_2_ moles originally injected into the headspace (based on the ideal gas law, this is 5.31 × 10^5^ nmoles C_2_H_2_). A template for calculating C_2_H_4_ production and rate of nitrogenase activity from GC output is provided (Supplementary File 1).

### Bacterial Strains

Bacterial strains used in this study are listed in Table 1. *Escherichia coli* and *Klebsiella oxytoca* were cultured on LB agar (Bertani, 1951), *Burkholderia vietnamiensis* was cultured on TY agar (Beringer, 1974) and the remaining strains were cultured on UMS agar (Poole et al., 1994) with 300 μM nicotinate, 10 mM NH_4_Cl_2_ as a sole nitrogen source, and either 30 mM malate (for *Azoarcus olearius, Azospirillum brasilense*, and *Herbaspirillum seropedicae*) or 20 mM succinate (for *Azorhizobium caulinodans* ORS571, hereby referred to as *Ac*) as a sole carbon source. All strains were grown at 28°C, except for *Ac* which was cultured at 37°C.

**TABLE 1:** Bacterial strains used in this study.

**Strain**	**Description**	**Origin**
*Azoarcus olearius* DQS-4	Betaproteobacterium, isolated from oil-contaminated soil in Taiwan.	Chen et al., 2013
*Azorhizobium caulinodans* ORS571	Alphaproteobacterium, isolated from *Sesbania rostrata* stems.	Dreyfus et al., 1988
*A. caulinodans Ac*LP	ORS571 harboring mini-Tn*7 attB* integration site from *Rhizobium* stably integrated into the chromosome.	This study
*A. caulinodans Ac*Cherry	*Ac*LP harboring a mini-Tn*7*-Gm cassette with *mCherry* expressed from the constitutive promoter J23104	This study
*Azospirillum brasilense* FP2	Alphaproteobacterium, spontaneous St^*R*^ mutant of Sp7 which was isolated from Tropical grasses in Brazil.	Tarrand et al., 1978
*Burkholderia vietnamiensis* WPB	Betaproteobacterium, isolated from *Populus* (cottonwood).	Doty et al., 2005
*Herbaspirillum seropedicae* SmR1	Betaproteobacterium, spontaneous St^*R*^ mutant of Z78 which was isolated from *Sorghum bicolor* in Brazil.	Baldani et al., 1996
*Klebsiella oxytoca* M5a1	Gammaproteobacterium, human pathogen isolated from soil.	Hamilton and Wilson, 1955
*Pseudomonas stuzeri* A1501	Gammaproteobacterium, isolated from the rice rhizosphere in southern China.	Qui et al., 1981
*Rhizobium leguminosarum* bv. v*iceae* 3841	Spontaneous Sm^*R*^ mutant of strain *R. leguminosarum* 300, symbiont of pea	Johnston and Beringer, 1975
*Rhodobacter sphaeroides* WS8	Alphaproteobacterium, isolated from soil in Ithica, NY, United States.	Clayton and Clayton, 1972

Plasmids were introduced into bacteria through diparental or triparental conjugation with *E. coli* ST18 donors (Thoma and Schobert, 2009) as previously described (Haskett et al., 2016). To construct strain *Ac*LP (Supplementary Figure 1), plasmid pOPS1475 harboring the *Rhizobium leguminosarum* biovar *viceae* 3841 Tn*7 attB* site was introduced into *Ac* and sucrose selection was used stably integrate this cargo into a harbor site in the chromosome by homologous recombination. To create strain *Ac*Cherry, a constitutively expressed *mCherry* gene carried on the mini-Tn*7* delivery plasmid pOPS1531 (Geddes et al., 2019b) was integrated into the Tn*7 attB* site of *Ac*LP following triparental conjugation with an *E. coli* ST18 carrying the transposase expression vector pTNS3 (Choi and Schweizer, 2006; Choi et al., 2008). Antibiotics for plasmid maintenance and selection were used at the following concentrations (μg mL^–1^): kanamycin 50 (*E. coli*) and 100 (*Ac*), carbenicillin 100, gentamycin 10 (*E. coli*), and 25 (*Ac*).

### Plasmids

Primers and Plasmids used in this study are listed in Supplementary Table 1 and Table 2, respectively. To construct pOPS1475, a 282-bp region of genomic DNA adjacent to *glmS* comprising the *Rhizobium leguminosarum* biovar *viceae* 3841 Tn*7 attB* site was amplified (oxp3374-75) and assembled with 1-kb flanking regions of DNA amplified from a harbor site in the *Ac* chromosome (primers oxp3372-73 and oxp3375-76) into pK19mobSacB digested SmaI using NEB HiFi assembly master-mix. Plasmid pOPS1213 was constructed by amplifying a 431-bp region of DNA capturing the *Ac nifH* promoter and RBS (primers oxp0104-05) and assembling this together with *sfGFP* (pOGG037) and the DT16 terminator (pOGG157) into the destination vector pOGG024 using modular golden-gate cloning with the type II restriction enzyme enzyme BsaI (NEB) (Weber et al., 2011; Geddes et al., 2019a). The resulting P*nifH*::*gfp*-DT16 cassette was amplified (oxp4793-94) and assembled using HiFi master-mix into the stable, broad-host-range plasmid pMQ131-PAR digested with ScaI. The current sequence of plasmid inserts were confirmed by Sanger sequencing.

**TABLE 2:** Plasmids used in this study.

**Plasmid**	**Description**	**Source**
pK19mobSacB	Suicide vector in rhizobia (R6K replicon) carrying SacB, Km^*R*^	Schäfer et al., 1994
pMQ131-PAR	Broad host-range (pBBR1 replicon) cloning plasmid with PAR genes, Km^*R*^	Guo et al., 2018
pOGG024	Broad host-range (pBBR1 replicon) BEVA destination vector, Gm^*R*^	Geddes et al., 2019a
pOGG037	BEVA plasmid harboring *sf*GFP SC-module, Sp^*R*^	Geddes et al., 2019a
pOGG157	BEVA plasmid harboring DT16 terminator T-module, Sp^*R*^	Geddes et al., 2019a
pOPS1213	pOGG024 carrying P*nifH::gfp*-DT16 reporter cassette, Gm^*R*^	This study
pOPS1475	pK19mobSacB vector carrying Tn*7 attB* integration site from *Rlv*3841 for integration into the *Ac*ORS571 chromosome, Km^*R*^	This study
pOPS1531	Mini-Tn*7* plasmid with *mCherry* expressed from the constitutive promoter J23104, Gm^*R*^, Ap^*R*^	Geddes et al., 2019b
pOPS1775	pMQ131-PAR with P*nifH*::GFP reporter cassette, Km^*R*^	This study
pTNS3	Transposase delivery plasmid for integration of mini-Tn*7* cassettes, Ap^*R*^	Choi and Schweizer, 2006

### Recovery of Bacteria and Viable Cell Counts

For estimation of combined bacterial population sizes occupying the *in situ* ARA vessels, 25 mL of PBS was added to the Schott bottles and vigorously agitated for 30 s. Viable counts were performed by establishing (five technical replicates per biological replicate) 10-fold serial dilutions of the resulting homogenous bacterial suspension from each Schott bottle and spotting 50 μL aliquots on non-selective agar plates. Colony morphology was examined to confirm that cross-contamination had not occurred, and the total number of cfu present was estimated based on the total volume (60 mL). No cfus were observed for drop counts performed on uninoculated control plants in this study.

Population sizes of *Ac*Cherry (pOPS1775) occupying the “root associated” (RA) fraction of *in situ* ARA vessels were isolated by uprooting barley plants and vortexing the excised root in PBS to remove loosely attached bacteria. After measuring the washed root fresh weights, roots were crushed with a sterile mortar and pestle and resuspended in 5 mL of PBS. The “rhizosphere” (RS) fraction was isolated by flushing the remaining sand with 10 mL of PBS and vigorously agitating for 30 s. Viable cell counts from the two fractions were performed as above on selective UMS agar media and total population estimations were calculated based on the total volumes of each fraction.

### Flow-Cytometry

Root associated and RS fractions of *Ac*Cherry (pOPS1775) were each centrifuged at 1,000 *g* and the supernatant was passed through a sterile 40 μm filter to clear growth substrate and plant material. RS fractions were subsequently diluted 10-fold with PBS before dispensing 80 μL aliquots of all samples into 96-well plates for analysis. To perform flow-cytometry, an Amnis^®^ Cellstream^®^ instrument with autosampler equipped with 488 and 561 nm lasers to excite GFP and mCherry, respectively, was used. Flow rates were set to high (14.64 μL/min) and at least 75,000 events defined by our gating parameters as “bacteria” were counted for each sample (Supplementary Figure 2). Using the CellStream^®^ Analysis 1.3.382 software, we gated for singlets based on the area (FSC) and aspect ratio (SSC), then gated cells exhibiting mCherry fluorescence (emission detected at 611/31 nm) above 5,000 fluorescence intensity (FI) units. The resulting population of bacterial cells was analyzed for mean mCherry and GFP fluorescence intensity (emission detected at 528/46 nm) and the ratio was calculated for each sample to give a standardized value for P*nifH::gfp* expression. Precise counts of gated bacteria at each step and mean FI values are given in Supplementary Table 2 and the data files for the experiment were uploaded to FlowRepository https://flowrepository.org/ (experiment FR-FCM-Z3QP).

### Confocal Microscopy

Dual channel confocal images were taken of whole lateral roots of barley using a ZEISS LSM 880 Axio Imager 2 with a C-Apochromat 40x/1.2W Korr FCS M27 objective. Excitation of GFP and mCherry was achieved using 488 (3% power) and 561 (4% power)nm lasers, respectively, and fluorescence emissions were collected using photomultiplier tube (PMT) detectors for GFP (493–598 nm, gain 500) and mCherry (detection 598–735, gain 600). Z-stack images were captured in 0.5 μm slices with the pinhole set to 1.39 AU using Line Sequential unidirectional scan mode. Maximum intensity projections were created from Z-stacks using the Zen 3.2 Blue software and are representative of 15 projections imaged from three plants (five per plant) for each treatment.

### Statistical Analysis

All statistical analyses were performed using the agricolae and RStatix packages in R (R Core Team, 2021). Relevant information regarding each statistical test is provided in the figure captions.

## Results

### Detection of Bacterial Nitrogenase Activity by *in situ* ARA

**FIGURE 2:**
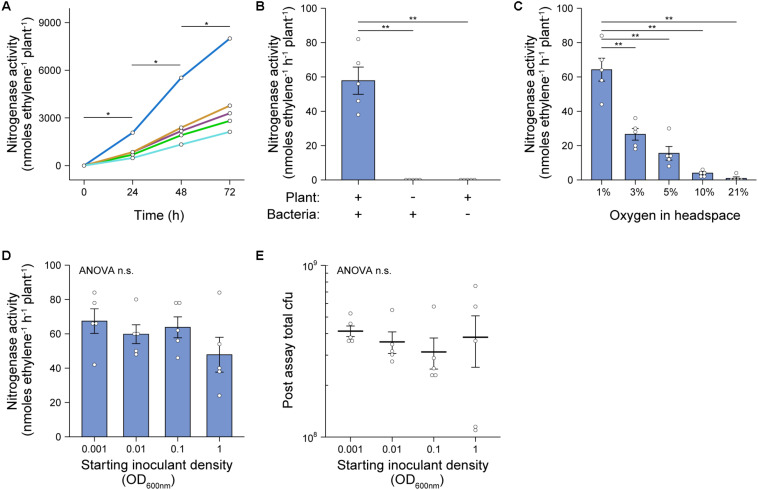
Validation and optimization of *in situ* ARA. *A. caulinodans* ORS571 (*Ac*) was used as the model strain for these experiments. The assays were set up by sewing individual pre-germinated, surface-sterilized barley seeds into 100 mL Schott bottles containing industrial grade sand and N/C-free rooting solution, inoculating the plants with 2 mL of an OD_600 nm_ 0.1 suspension of *Ac*, then growing the plants in a growth-chamber for 6-days. At this point, the atmosphere in the headspace was adjusted to 1% O_2_, the bottles were sealed with a rubber septum and 10% of the headspace was replaced with C_2_H_2_. Bottles were returned to the growth chamber and the reduction of acetylene (C_2_H_2_) to ethylene (C_4_H_4_) by nitrogenase was measured at 24-intervals over 72-h using GC-MS. Mean ± SEM (error bars) and individual values for five biological replicates are plotted. Nitrogenase activity was calculated between 24-h and 48-h. ANOVA and pairwise two-tailed students *t*-tests with Bonferroni adjusted *P*-values were used to compare means where relevant. *P* > 0.05 not significant (n.s.), *P < 0.05, **P < 0.01, ***P < 0.001. **(A)** C_2_H_4_ production in each of five biological replicates was monitored over 72-h. **(B)** Rates of nitrogenase activity where measured when the plant or bacteria was omitted from the system. **(C)** Rates of nitrogenase activity were measured when the starting O_2_ concentration in the headspace was adjusted between 1 and 21% (i.e., air) by flushing with N_2_ gas. **(D)** Rates of nitrogenase activity were measured when the starting inoculant density was adjusted between OD_600 nm_ 0.001 to 1. **(E)** Total colony forming units (cfu) present in the assay systems of experiment **(D)** as determined by viable counts after 72-h.

To both validate and optimize our *in situ* ARA (Figure 1), we utilized the cereal endophyte and *Sesbania rostrata* nodulating symbiont *Azorhizobium caulinodans* ORS571 (*Ac*) as a model diazotroph and barley as the model host cereal. The assay was initially set up by sewing individual pre-germinated, surface-sterilized Barley seeds into 100 mL Schott bottles containing industrial grade fire sand and N/C-free rooting solution, inoculating the plants with 2 mL of an OD_600 nm_ 0.1 suspension of *Ac* (approximately 5 × 10^7^ cells), then growing the plants in a growth-chamber for 6-days. At this point, the atmosphere in the headspace was adjusted to 1% O_2_ and Schott bottles were sealed with a rubber septum. Ten percent of the headspace was next replaced with C_2_H_2_ and plants were returned to the growth chamber. The reduction of C_2_H_2_ to C_4_H_4_ by nitrogenase was measured over 72-h using GC. In all five biological replicates, significant C_2_H_4_ production (mean 69.97 ± SEM 17.05 nmoles C_2_H_4_h^–1^ plant^–1^) was detected after 24-h and the subsequent rates of C_2_H_4_ production remained stable up to 72-h with a mean rate of nitrogenase activity of 55.57 ± SEM. 11.23 nmol C_2_H_4_ h^–1^ plant^–1^ between 24 and 48-h, and 62.77 ± SEM. 14.11 nmol C_2_H_4_ h^–1^ plant^–1^ between 48 and 72-h (Figure 2A). Using the above conditions, we also confirmed that neither the plant nor bacteria exhibited nitrogenase activity in the absence of the symbiotic partner over 72-h (Figure 2B). Thus, bacterial nitrogenase activity monitored in the assay was entirely dependent on nutrients provided by the host plant.

### Effects of Titrating O_2_ Concentration and Starting Inoculant Density

We further optimized our *in situ* ARA first by titrating the starting O_2_ concentration in the headspace of Schott bottles after 6-dpi. We found that an optimum rate of nitrogenase activity, similar to that of the experiments in Figures 2A,B, was observed where O_2_ in the headspace was adjusted to 1% of the atmosphere (Figure 2C). A low rate of nitrogenase activity (mean 3.98 ± SEM. 0.83 nmol C_2_H_4_ h^–1^ plant^–1^) was also observed where the O_2_ concentration was adjusted to 10%. We also tested the effect of titrating the starting inoculant density between OD_600 nm_ 0.001 and 1.0 and found that nitrogenase activity did not differ between these treatments (Figure 2D). Moreover, when bacteria were recovered at the close of the assay by rigorous flushing with PBS, the total number of colony-forming units (cfu) regrown from each Schott bottle did not differ significantly, with each harboring between 10^8^ and 10^9^ cfu (Figure 2E). This suggested that after 6-dpi, *Ac* naturally reaches the carrying capacity of the assay system independently of the starting inoculation density.

### Contribution of Rhizosphere and Root-Associated Bacterial Nitrogenase Expression

**FIGURE 3:**
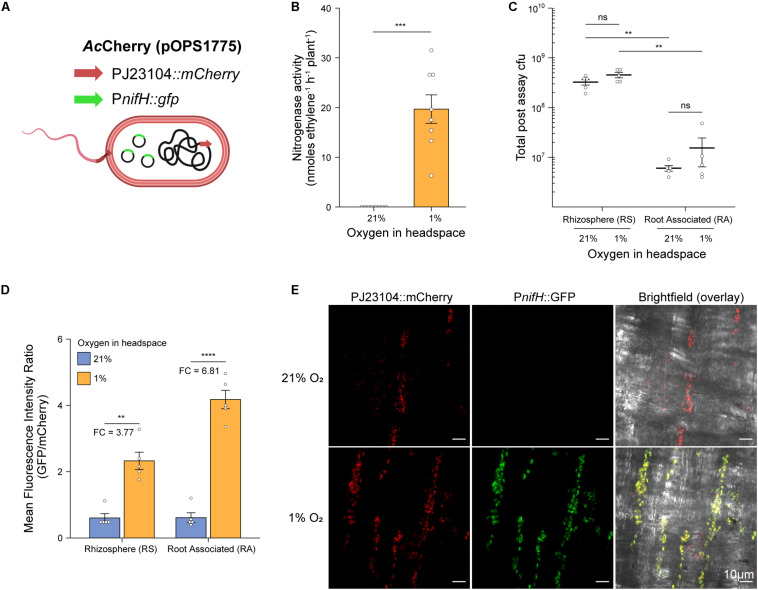
Rhizosphere and root associated nitrogenase expression during *in situ* ARAs. To assess spatial expression of nitrogenase in our *in situ* ARA systems, **(A)** we marked the chromosome of strain *Ac*LP with a constitutively expressed mCherry cassette derived from pOPS1531 and introduced into the resulting strain (*Ac*Cherry) plasmid pOPS1775 which carried the promoter of the nitrogenase structural gene *nifH* fused to a GFP reporter. It was previously shown that expression from P*nifH* is induced under N-fixing (low O_2_ and N) conditions (Kaminski and Elmerich, 1991; Michel-Reydellet and Kaminski, 1999), thus comparison of GFP fluorescence in *in situ* ARAs performed with a headspace of 1% vs. 21% could be used to assess *nifH* expression. **(B)** Mean ± SEM (error bars) rates of nitrogenase activity derived from eight *in situ* ARAs of *Ac*Cherry (pOPS1775) inoculated at OD_600 nm_ 0.1 onto barley. **(C)** Estimated population sizes of bacteria occupying the rhizosphere and soil (RS) or rhizoplane and endosphere (root associated, RA) from five plants on which *in situ* ARAs were performed. **(D)** Comparison of standardized P*nifH::gfp* expression (mean GFP/mCherry fluorescence intensity ratio) by flow-cytometry of bacteria occupying the RS and RA fractions from the same five plants in which *in situ* ARAs were performed. Values for fold-changes (FC) in expression are provided **(E)** Maximum intensity projections showing mCherry and GFP fluorescence by *Ac*Cherry (pOPS1775) cells colonizing the rhizoplane of plants on which *in situ* ARAs were performed. Each image is representative of 15 images acquired from five whole lateral roots excised from three plants per treatment. ANOVA and two-tailed student’s *t*-tests with Bonferroni adjusted *P*-values were used to compare means where relevant. *P* > 0.05 not significant (n.s.), *P < 0.05, **P < 0.01, ***P < 0.001, and ****P < 0.0001. Panel A was created using BioRender^®^.

To explore the spatial patterns of colonization and nitrogenase expression by bacteria in our *in situ* ARAs, we designed a dual reporter *Ac* strain stably marked with a constitutively expressed *mCherry* reporter gene (strain *Ac*Cherry) which additionally carried the promoter of the nitrogenase structural gene *nifH* fused to a *GFP* reporter gene on the broad host-range plasmid pOPS1775 (Figure 3A). Because the P*nifH* promoter is induced under N-fixing conditions (low O_2_ and N) by the master regulator NifA (Kaminski and Elmerich, 1991; Michel-Reydellet and Kaminski, 1999), comparison of GFP fluorescence in bacteria isolated from *in situ* ARAs performed with 1% and 21% O_2_ in the headspace could be used to assess spatial expression of nitrogenase. We initially confirmed that strain *Ac*Cherry carrying pOPS1775 exhibited nitrogenase activity when *in situ* ARAs were performed with a headspace of 1% oxygen and starting inoculation density of OD_600 nm_ 0.1 (Figure 3B), although this was suboptimal (mean 19.69 ± SEM. 2.87 nmol C_2_H_4_ h^–1^ plant^–1^) compared to the wild-type in previous experiments (Figures 2A–D) presumably due to titration of NifA by the multi-copy plasmid-borne P*nifH* promoter and (or) increased energy demand due to expression of the fluorescent proteins. As expected, nitrogenase activity for *Ac*Cherry (pOPS1775) was not observed when the headspace contained 21% O_2_ (Figure 3B).

Following *in situ* ARAs, *Ac*Cherry (pOPS1775) cells were isolated from the root surface and endosphere, here termed RA fraction, and from the rhizosphere and surrounding soil (RS fraction) and viable counts were performed to estimate total population sizes (Figure 3C). No significant differences were observed in the total population sizes when comparing the same fraction between O_2_ treatments. However, for both O_2_ treatments, the RS fraction harbored an order of magnitude more cfus then did the RA fraction.

We next assessed expression of the P*nifH*::gfp reporter fusion carried by *Ac*Cherry (pOPS1775) in the RS and RA fractions by using flow-cytometry to compare the mean fluorescence intensity (MFI) ratio of GFP to mCherry for single bacterial cells identified based on positive mCherry expression above 5000 fluorescence units (Supplementary Figure 2 and Figure 3D). We found that the MFI ratio for both fractions of bacteria isolated from the 21% O_2_ treated *in situ* ARAs was approximately 0.6, while the MFI ratio for RS and RA fractions isolated from the 1% O_2_ treatments was increased 3.77-fold and 6.81-fold, respectively, indicating induction of nitrogenase expression. We also performed confocal microscopy on lateral roots excised from three additional plants that were subject to *in situ* ARAs and confirmed visually the induction of the P*nifH::gfp* cassette under 1% O_2_ relative to 21% (Figure 3E). Overall, these experiments demonstrate that both the plant-associated bacteria attached to the root and bacteria occupying the rhizosphere contribute to nitrogenase expression during *in situ* ARAs.

### Demonstration of *in situ* ARA on Eight Genetically Diverse Diazotrophs

**FIGURE 4:**
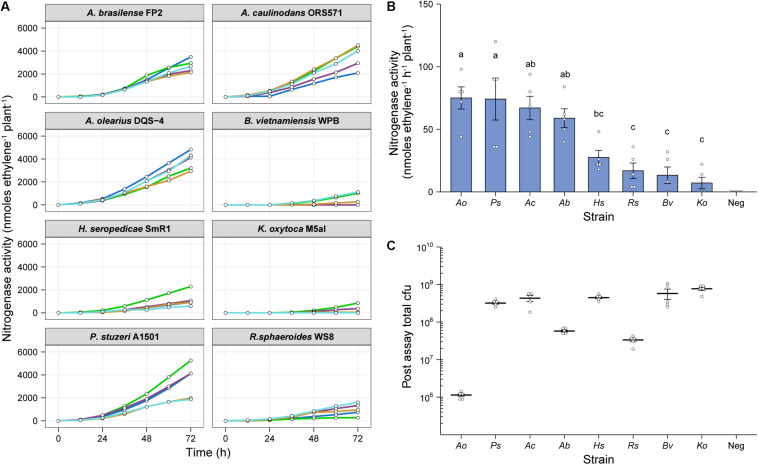
*In situ* ARA of eight model diazotrohic bacteria. Assays were set up as described in Figure 2. Two milliliters of an OD_600 nm_ 0.01 suspension of bacteria were used for inoculation and C_2_H_4_ production was measured at 12-h intervals over 72-h. **(A)** C_2_H_4_ production was measured for five biological replicates of eight genetically diverse diazotrophic bacteria inoculated gnotobiotically onto barley. **(B)** The mean ± SEM (error bars) rates of nitrogenase activity between 48-h and 62-h were calculated for the eight model diazotrophs. ANOVA and LSD tests with Bonferroni adjusted *P*-values were used to compare means. The negative uninoculated control omitted from statistical tests. Matching letters depict treatments that are not significantly different (*P* > 0.05) from each other but are significantly different (*P* < 0.05) from treatments with a distinct letter. **(C)** Mean ± SEM (error bars) of total cfu present in the assay systems as determined by viable counts after 72-h. No cfu were observed for the uninoculated control. Note that the low total cfu count for *Azoarcus olerarius* DQS-4 may be a result of poor viability of the strain upon re-isolation from the plant, as has been reported previously (Hurek et al., 2002).

To test whether our *in situ* ARA could be used to assess nitrogenase activity by diazotrophs other than *Ac*, we selected the following seven additional alpha-, beta- and gamma-proteobacterial strains for testing; *Azospirillum brasilense* FP2 (*Ab*), *Azoarcus olearius* DQS-4 (*Ao*), *Burkholderia vietnamensis* WPB (*Bv*), *Herbaspirillum seropedicae* SmR1 (*Hs*), *Klebsiella oxytoca* M5a1 (*Ko*), *Pseudomonas stuzeri* A1501 (*Ps*), and *Rhodobacter sphaeoroides* WS8 (*Rs*). Plants were inoculated with 2 mL of an OD_600 nm_ 0.01 suspension and after 6-dpi, the atmosphere in the headspace was adjusted to 1% O_2_ and 10% C_2_H_2_ to begin the assay. GC measurements for C_2_H_4_ production were made at 12-h intervals over 72-h. For most of the strains, nitrogenase activity was detectable by 24-h, but a stable, optimal rate of nitrogenase activity required at least 36-h of incubation (Figure 4A). Mean rates of nitrogenase activity were measured for all strains between 48 and 62-h (Figure 4B), with the highest rates between 58 and 65 nmol C_2_H_4_ h^–1^ plant^–1^ for *Ao, Ps, Ac*, and *Ab.* The remaining strains fixed between 7 and 30 nmol C_2_H_4_ h^–1^ plant^–1^, whereas no nitrogenase activity was detected in the uninoculated controls.

We next recovered bacteria from the assays and performed viable drop counts to estimate the total number of cfu in each treatment. Interestingly, *Ao*, which exhibited the highest mean nitrogenase activity, was found to be the least abundant in the barley root systems (Figure 4C), indicating that individual cells may be capable of fixing a considerable amount of N relative to the other strains. However, we suspect that this result may be influenced by poor viability of the bacterium upon recovery from the plant, as has previously been documented (Hurek et al., 2002). Conversely, the strains which were highly abundant in the barley root system, *Bv* and *Ko*, exhibited comparatively poor nitrogenase activity, suggesting that these strains are poorly adapted to N fixation under these given set of experimental conditions.

## Discussion

We have demonstrated that our simple *in situ* ARA is highly reproducible, rapid (can be completed in under 2-weeks) and is applicable to a diverse range of diazotrophic bacteria. The simple standardization involved in our assay workflow (i.e., nitrogenase activity per plant) is one of its most beneficial features, offering a reduced workload compared to other potential approaches. We observed that the total population size of *Ac* in the assay system reached equilibrium after 9-dpi regardless of the starting inoculation density (Figure 2E), and that N fixation was consistent between these treatments (Figure 2C). Nevertheless, there was some variation observed in the total population size when comparing eight genetically diverse endophytes for N fixation (Figure 4). Therefore, in some instances it could be useful to standardize measurements of nitrogenase on a per cell basis. We propose that fluorescent labeling of bacteria in combination with confocal microscopy or flow-cytometry (Bloemberg, 2007) would be a suitable strategy if rates need to be expressed per cell (Figures 2E, 4C) because some bacteria, such as *Ao*, exhibit poor viability upon recovery from plants (Hurek et al., 2002) which could obscure measurements. Dual fluorescent reporter gene fusions could also be made to promoters of the nitrogenase structural gene *nifH* to assess the spatiotemporal dynamics of nitrogenase expression as we have done for *Ac* (Figure 3) and has been used in the past for other diazotrophic bacteria (Egener et al., 1998, 1999; Roncato-Maccari et al., 2003; Santos et al., 2017).

One of the major constraints of measuring plant associative nitrogenase activity and N fixation is that optimal nitrogenase activity for most bacteria requires environmental O_2_ concentrations below 21% which may be detrimental for photosynthesis and plant growth (Priestley et al., 1988). Although long-term exposure of plants to low oxygen ultimately results in anoxia, leading to acidosis and apoptosis, plants are able to postpone or even prevent tissue from becoming anoxic by tuning the expression or activity of energetically demanding metabolic pathways (Geigenberger et al., 2000; Geigenberger, 2003; Zabalza et al., 2009) and by producing non-symbiotic leghaemoglobins that help maintain redox status and remove reactive oxygen and nitrogen species (Igamberdiev et al., 2014). Remarkably, in our assay system, nitrogenase expression was detected in bacteria attached to the root and occupying the rhizosphere (Figure 3), and nitrogenase activity was stable over a 72-h period for all headspace O_2_ concentrations tested (Figure 2C), indicating that the plant is still able to provide adequate nutrients to fuel bacterial N fixation under these conditions. In addition to carbon, sustained production of signaling molecules would also be critical to permit nitrogenase expression and activity in engineered bacteria that activate nitrogenase activity in response to exogenously added plant-derived signaling molecules in free-living culture conditions (Ryu et al., 2020). Testing such strains using our assay system will be pivotal to the development of such strains for environmental use.

In this work, we utilized barley as a host plant due to the highly uniform growth characteristics of seedings, but also due to its status as a model cereal for engineering the capacity for N fixation^1^ and the availability of a sequenced genome (Mascher et al., 2017). We suspect that the assay could be readily extended to compare nitrogenase activity in bacteria colonizing other host plants, however, this may require additional standardization to account for differences in plant root mass. On the same note, the assay could be readily extended to assess the influence of various abiotic factors, such as plant growth substrates, O_2_, nutrients, pollutants, temperature, or light, or be used to explore the influences of abiotic factors on nitrogenase activity. The latter could be achieved for example by performing co-inoculation assays or performing *in situ* ARAs with non-sterile field soils, although this might be partially impeded by the presence of native N-fixing bacteria. Alternatively, defined synthetic communities of bacteria (Grosskopf and Soyer, 2014) could be inoculated as competitors for the diazotroph of interest. The validation and optimization of our assay presented here has paved the way for such future extensions.

## Data Availability Statement

The research materials supporting this publication can be accessed by contacting TK, tim.haskett@plants.ox.ac.uk.

## Author Contributions

TH and MM conceptualized the experiments and analyzed the data. TH, HK, BJ, and MM performed the experiments. PP provided supervision and equipment for the experiments. TH and PP contributed to editing of the manuscript. All authors contributed to the article and approved the submitted version of the manuscript.

## Conflict of Interest

The authors declare that the research was conducted in the absence of any commercial or financial relationships that could be construed as a potential conflict of interest.

## Footnotes

1 https://www.ensa.ac.uk/

## Supplementary Material

Supplementary Figure 1 Construction of strain *Ac*LP. To construct *Ac*LP, a 282-bp region of genomic DNA adjacent to *glmS* comprising the *Rhizobium leguminosarum* biovar *viceae* 3841 Tn*7 attB* site was amplified and assembled with 1-kb flanking regions of DNA amplified from a harbor site in the *Ac* chromosome into pK19mobSacB, creating plasmid pOPS1475. Plasmid pOPS1475 was introduced into *Ac* and sucrose selection was used stably integrate the cargo into the *Ac* chromosome by homologous recombination.

Supplementary Figure 2 Flow-cytometry gating strategy to assess P*nifH* expression by bacteria re-isolated from *in situ* ARAs. Using the CellStream^®^ Analysis 1.3.382 software, we defined an arbitrary gating parameter for “bacteria,” then gated for singlets within this population based on the area (FSC) and aspect ratio (SSC). Singlets exhibiting mCherry fluorescence (emission detected at 611–31 nm) above 5,000 fluorescence intensity (FI) units were gated and mean mCherry and GFP (emission detected at 528–46 nm) fluorescence intensity ratios were analyzed within this population.

Supplementary Table 1 Primers used in this study.

Supplementary Table 2 Flow-cytometry cell counts and mean fluorescence intensity (MFI) values.

Supplementary File 1 Template for calculating C_2_H_4_ production and nitrogenase activity from GC output.
