# Supplementary figures and images for: A Simple in situ Assay to Assess Plant-Associative Bacterial Nitrogenase Activity
Source: Front Microbiol. 2021 Jun 23;12:690439. doi: 10.3389/fmicb.2021.690439 (PMC8261070; doi:10.3389/fmicb.2021.690439)

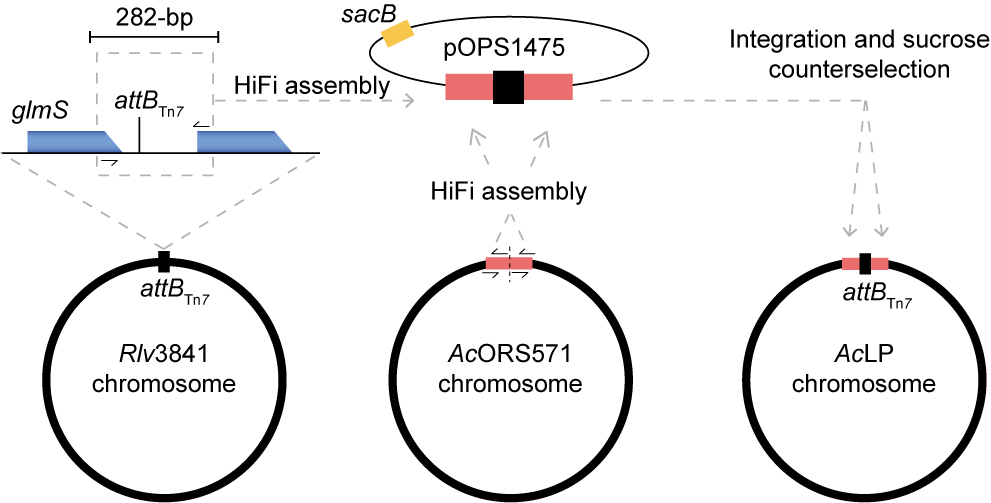

Supplement: Supplementary Figure 1 — Construction of strain AcLP. To construct AcLP, a 282-bp region of genomic DNA adjacent to glmS comprising the Rhizobium leguminosarum biovar viceae 3841 Tn7 attB site was amplified and assembled with 1-kb flanking regions of DNA amplified from a harbor site in the Ac chromosome into pK19mobSacB, creating plasmid pOPS1475. Plasmid pOPS1475 was introduced into Ac and sucrose selection was used stably integrate the cargo into the Ac chromosome by homologous recombination. [file Image_1.tif]

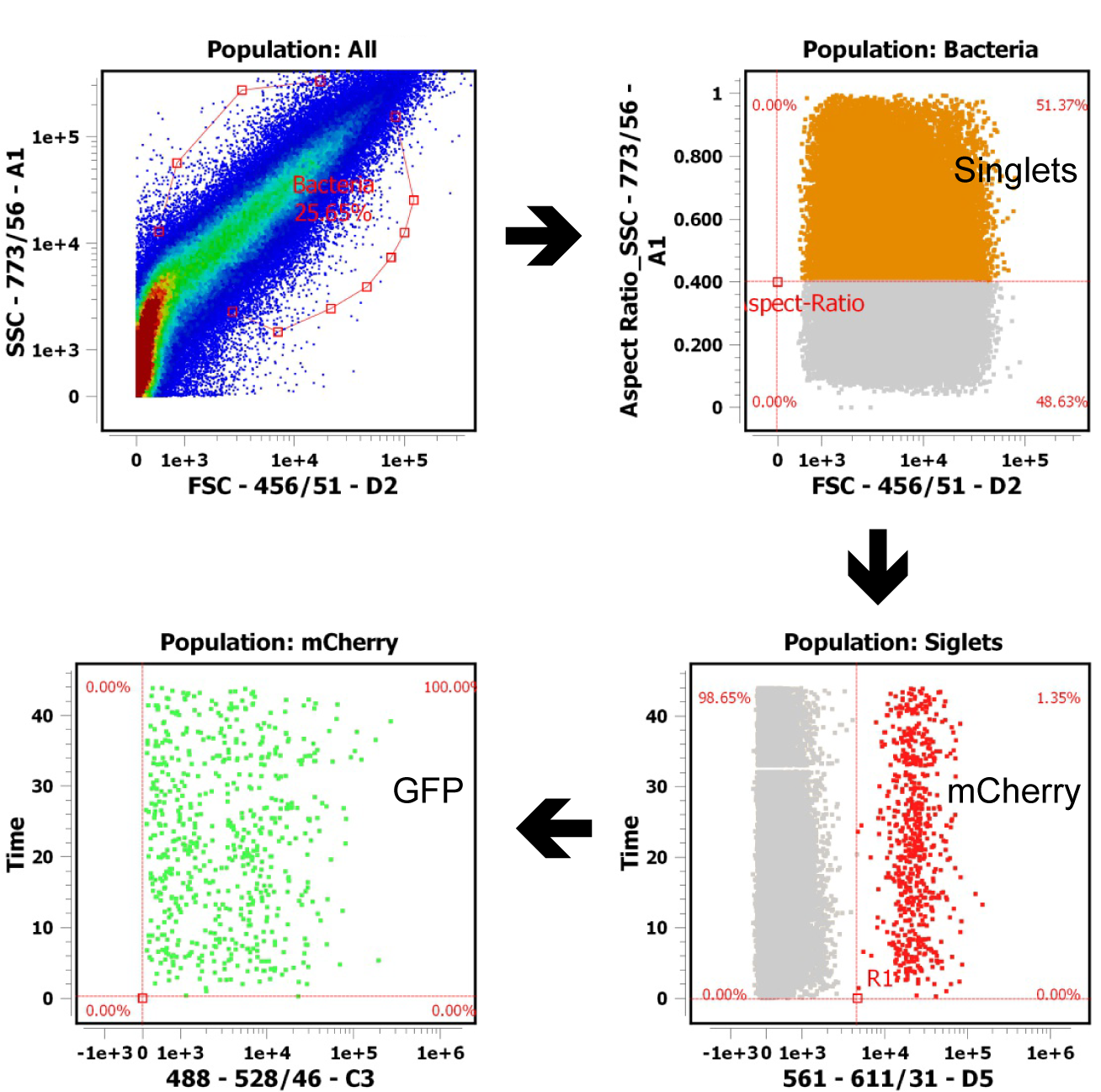

Supplement: Supplementary Figure 2 — Flow-cytometry gating strategy to assess PnifH expression by bacteria re-isolated from in situ ARAs. Using the CellStream® Analysis 1.3.382 software, we defined an arbitrary gating parameter for “bacteria,” then gated for singlets within this population based on the area (FSC) and aspect ratio (SSC). Singlets exhibiting mCherry fluorescence (emission detected at 611–31 nm) above 5,000 fluorescence intensity (FI) units were gated and mean mCherry and GFP (emission detected at 528–46 nm) fluorescence intensity ratios were analyzed within this population. [file Image_2.tif]
