# Supplementary material for: A Simple in situ Assay to Assess Plant-Associative Bacterial Nitrogenase Activity
Source: Front Microbiol. 2021 Jun 23;12:690439. doi: 10.3389/fmicb.2021.690439 (PMC8261070; doi:10.3389/fmicb.2021.690439)
Supplement: Supplementary Table 1 — Primers used in this study. [file Table_1.docx]

**Table S1. Primers used in this study**

| **Primer name** | **Sequence** |
| --- | --- |
| oxp0104 | ATATGGATCCGGCAGCCTCGCTCGATGCGG |
| oxp0105 | ATATGGTACCGTCTTGAATTCCTTCGAACC |
| oxp3372 | GTCGACTCTAGAGGATCCCCTTGTTTTCGGGAGTTGGCTG |
| oxp3373 | GGTGTGATAGAAGCCTTCGCGGACAAGC |
| oxp3374 | GCGAAGGCTTCTATCACACCGCCGTCTTC |
| oxp3375 | AGCCTGACATATGATCAGCCCTGCGAGAAAATTATTC |
| oxp3376 | GGCTGATCATATGTCAGGCTGGCAGGTC |
| oxp3377 | TGAATTCGAGCTCGGTACCCCGGTTCAAAGCATGTCGAC |
| oxp4793 | TAATACAGTCAAATTGCAGTACAGGCTTATGTCAATTGCCTG |
| oxp4794 | TCTGTATACACCCGCAGAGTTGCCGAATTCGGATCCGG |
