# Supplementary material for: A Simple in situ Assay to Assess Plant-Associative Bacterial Nitrogenase Activity
Source: Front Microbiol. 2021 Jun 23;12:690439. doi: 10.3389/fmicb.2021.690439 (PMC8261070; doi:10.3389/fmicb.2021.690439)
Supplement: Supplementary Table 2 — Flow-cytometry cell counts and mean fluorescence intensity (MFI) values. [file Table_2.docx]

**Table S2. Flow-cytometry cell counts and mean fluorescence intensity (MFI) values**

| **Treatment** | **Plant** | **Fraction** | **Count (bacteria)** | **Count (Singlets)** | **Count (mCherry)** | **Count (GFP)** | **MFI (GFP)** | **MFI (mCherry)** |
| --- | --- | --- | --- | --- | --- | --- | --- | --- |
| 1% O2 | 1 | RA | 95279 | 49733 | 453 | 453 | 19318.6 | 4139.8 |
| 1% O2 | 2 | RA | 89972 | 46734 | 632 | 632 | 9162.7 | 2718.7 |
| 1% O2 | 3 | RA | 75751 | 40004 | 407 | 407 | 11987.2 | 3033.3 |
| 1% O2 | 4 | RA | 70658 | 37592 | 317 | 317 | 21995.1 | 4457.7 |
| 1% O2 | 5 | RA | 76843 | 40299 | 280 | 280 | 12571.5 | 3171.2 |
| 1% O2 | 1 | RS | 97952 | 50009 | 285 | 285 | 8812.5 | 2703.7 |
| 1% O2 | 2 | RS | 103388 | 52892 | 495 | 495 | 4142.1 | 2037.7 |
| 1% O2 | 3 | RS | 103503 | 53756 | 238 | 238 | 3580 | 2060.4 |
| 1% O2 | 4 | RS | 102775 | 52666 | 335 | 335 | 4657.2 | 2139.4 |
| 1% O2 | 5 | RS | 97880 | 49865 | 251 | 251 | 4911.4 | 2016.6 |
| 21% O2 | 6 | RA | 86948 | 45333 | 196 | 196 | 859.4 | 2065.9 |
| 21% O2 | 7 | RA | 90412 | 46863 | 93 | 93 | 1032.9 | 1946.4 |
| 21% O2 | 8 | RA | 90366 | 46979 | 133 | 133 | 887.7 | 1868.3 |
| 21% O2 | 9 | RA | 110871 | 57077 | 178 | 178 | 937.9 | 2065.6 |
| 21% O2 | 10 | RA | 110724 | 56672 | 171 | 171 | 2694.8 | 2263.7 |
| 21% O2 | 6 | RS | 88287 | 45351 | 139 | 139 | 752.9 | 1642.3 |
| 21% O2 | 7 | RS | 100111 | 51707 | 175 | 175 | 840.7 | 1697.7 |
| 21% O2 | 8 | RS | 102567 | 53489 | 313 | 313 | 805.1 | 1653.7 |
| 21% O2 | 9 | RS | 105064 | 52900 | 360 | 360 | 783.4 | 1702.1 |
| 21% O2 | 10 | RS | 99013 | 50348 | 561 | 561 | 2155 | 1919.7 |
